# *CsPAO4* of *Citrus sinensis* functions in polyamine terminal catabolism and inhibits plant growth under salt stress

**DOI:** 10.1038/srep31384

**Published:** 2016-08-18

**Authors:** Wei Wang, Ji-Hong Liu

**Affiliations:** 1 Key Laboratory of Horticultural Plant Biology, MOE, College of Horticulture and Forestry Sciences, Huazhong Agricultural University, Wuhan 430070, China

## Abstract

Polyamine oxidase (PAO) is a key enzyme catalyzing polyamine catabolism leading to H_2_O_2_ production. We previously demonstrated that *Citrus sinensis* contains six putative *PAO* genes, but their functions are not well understood. In this work, we reported functional elucidation of *CsPAO4* in polyamine catabolism and salt stress response. CsPAO4 was localized to the apoplast and used both spermidine (Spd) and spermine (Spm) as substrates for terminal catabolism. Transgenic plants overexpressing *CsPAO4* displayed prominent increase in PAO activity, concurrent with marked decrease of Spm and Spd and elevation of H_2_O_2_. Seeds of transgenic lines displayed better germination when compared with wild type (WT) under salt stress. However, both vegetative growth and root elongation of the transgenic lines were prominently inhibited under salt stress, accompanied by higher level of H_2_O_2_ and more conspicuous programmed cell death (PCD). Exogenous supply of catalase (CAT), a H_2_O_2_ scavenger, partially recovered the vegetative growth and root elongation. In addition, spermine inhibited root growth of transgenic plants. Taken together, these data demonstrated that *CsPAO4* accounts for production of H_2_O_2_ causing oxidative damages under salt stress and that down-regulation of a *PAO* gene involved in polyamine terminal catabolism may be an alternative approach for improving salt stress tolerance.

Plants are constantly challenged by a variety of abiotic stresses, such as extreme temperatures, salt and drought, that impose negative influence on plant growth, development, and crop productivity. One common feature shared by these environmental stresses is the elevated generation of reactive oxygen species (ROS). The ROS, if not scavenged timely and efficiently, are toxic to plant cells as they can cause oxidative damages to proteins, DNA and lipids, implying that reduction of ROS levels is crucial for cellular survival under environmental stresses^1^,^2^. H_2_O_2_ is one of the major ROS that accounts for generation of oxidative stress and disruption of normal metabolism in response to adverse environmental cues. It is conceivable that inhibition of excessive H_2_O_2_ accumulation might act as an effective approach to alleviate ROS-associated injury, leading to improved stress tolerance^1^. Therefore, understanding of critical genes involved in production of H_2_O_2_ may provide valuable knowledge for genetically improving stress tolerance via modulation of cellular ROS levels. Several enzymatic reactions are responsible for generation of H_2_O_2_ under normal and stress conditions, one of which is mediated by oxidation of polyamines^3^,^4^,^5^. Polyamines (PAs) are low-molecular-mass nitrogenous compounds, and the most common PAs in plants are diamine putrescine (Put), triamine spermidine (Spd) and tetramine spermine (Spm)^6^. Increasing studies have reported that plant PAs are involved in tolerance to abiotic stresses, such as high/low temperature, salinity and drought. On one hand, the expression levels of several genes involved in PA biosynthesis and PAs contents are increased in the presence of one or more abiotic stresses^7^,^8^. Under salt stress, in most case Put level decreased while Spd and/or Spm levels increased in several plants, such as *Spinacia oleracea*, *Lactuca sativa*, *Cucumis melo* and *Oryza sativa*^9^. The capacity of plants to endure stresses is, to some extent, correlated to their ability to synthesize PAs^10^,^11^. On the other hand, exogenous supply of PAs can enhance abiotic stress tolerance while decrease endogenous PAs can weaken stress tolerance. For example, exogenous spermidine is enhancing tomato tolerance to salinity-alkalinity stress by regulating chloroplast antioxidant system and chlorophyll metabolism^12^. Downregulating the expression level of SAMDC could reduce PAs synthesis in tobacco and make the plants more sensitive to salt stress^13^. Recently, the ratio of PA catabolism and PA anabolism is suggested as the crucial factor in PA-mediated stress tolerance^14^.

The catabolism of PAs is catalyzed by two classes of amine enzymes, copper-containing amine oxidases (CuAOs) and FAD-dependent polyamine oxidases (PAOs)^15^. CuAOs mainly oxidize Put and cadaverine (Cad), producing 4-aminobutanal, H_2_O_2_ and ammonia^16^. In contrast to CuAOs, PAOs mainly oxidize Spd and Spm and can be divided into two groups based on their reaction modes^6^. The first group of PAOs, generally located in apoplast, catalyze the PAs terminal catabolism, yielding 1,3-diaminopropane (Dap), H_2_O_2_, and N-(3-aminopropyl)-4 -aminobutanal (Spm catabolism) or 4-aminobutanal (Spd catabolism)^15^,^17^. ZmPAO of maize is the best-characterized plant PAO involved in terminal catabolism of PAs^18^. PAOs of barley^19^ and rice (OsPAO7)^20^ are also proven to catalyze the terminal catabolism of PAs. The PAOs of second group, located in intracellular space like cytoplasm or peroxisome, catalyze PAs back conversion reactions, which convert Spm into Spd and Put or Spd into Put, producing H_2_O_2_ concomitantly. *Arabidopsis thaliana* genome contains five PAOs (AtPAO1-AtPAO5), and all of them catalyze back conversion of PAs^21^,^22^,^23^,^24^,^25^. Rice genome contains seven PAOs (OsPAO1-OsPAO7), in which four (OsPAO1, OsPAO3-OsPAO5) are identified to be involved in PAs back conversion reactions^26^,^27^. Recently, *PAO* genes involved in back conversion reactions of PAs were identified in cotton and citrus^28^,^29^.

Increasing studies suggest that PAs catabolism plays a critical role in many plant physiological progresses, such as plant development and stress response^6^. Rodríguez *et al.*^30^ demonstrated that PAO-mediated ROS source partially contributed to the maize leaf blade elongation. Wu *et al.*^31^ reported that H_2_O_2_ from Spd oxidation can signal Ca^2+^ influx and regulate pollen tube growth. Perturbation of PAs catabolism in tobacco strongly affected root development and xylem differentiation^32^. Recently, Kim *et al.*^33^ demonstrated that *AtPAO5* loss-of-function mutant (*pao5*) presented delayed transition from vegetative to reproductive growth. In addition, PAO is also associated with abiotic stress response. Moschou *et al.*^22^ reported that under salt stress Spd was secreted into apoplast and catabolized by PAO to produce H_2_O_2_, which further triggered additional protective stress responses or PCD. Furthermore, overexpression of *ZmPAO* in tobacco led to plant growth inhibition due to PCD induced by overproduction of H_2_O_2_ under salt stress.

Although H_2_O_2_ can be produced from PA oxidation via back conversion and terminal catabolism, so far knowledge concerning the role of H_2_O_2_ derived from PA terminal catabolism in stress tolerance remains poorly understood. In an earlier work, we identified six putative *PAO* genes in sweet orange (*Citrus sinensis*), and analyzed their expression profiles in response to abiotic stresses^29^. One of these members, *CsPAO4*, was postulated to be possibly involved in PAs terminal catabolism based on the expression pattern, but whether this postulation is right remains unanswered. Here, we first investigated subcellular localization of CsPAO4, and then generated transgenic plants overexpressing *CsPAO4* to examine its role in PAs catabolism and salt stress response. Our results showed CsPAO4 was localized in apoplast and played a role in terminal catabolism of PAs. Overexpression of *CsPAO4* promoted seed germination, but inhibited plant growth under salt stress, which is largely ascribed to PCD caused by excess production of PAO-derived H_2_O_2_. Taken together, our work demonstrates that *PAO* genes involved in terminal catabolism are critical for H_2_O_2_ accumulation under salt stress, which suggests that it can be considered as a target to be down-regulated for genetically improving salt stress tolerance.

## Result

### Alignment of CsPAO4 with other PAOs involved in PAs terminal catabolism

Our previous work suggested that CsPAO4 probably catalyzes the PAs terminal catabolism. In order to verify this, we first aligned the amino acid sequence of *CsPAO4* with other plant PAOs that have been reported to be implicated in PAs terminal catabolism. The result showed CsPAO4 shared low degree of sequence identity with other PAOs although some conserved regions existed among the examined PAOs. Different from the PAOs in other plants, CsPAO4 contained an transmembrane domain (TD), but no signal peptide (SP) was detected (Fig. S1). Nevertheless, CsPAO4 possessed all the putative residues associated with catalytic activity.

### CsPAO4 was localized in apoplast

**Figure 1:**
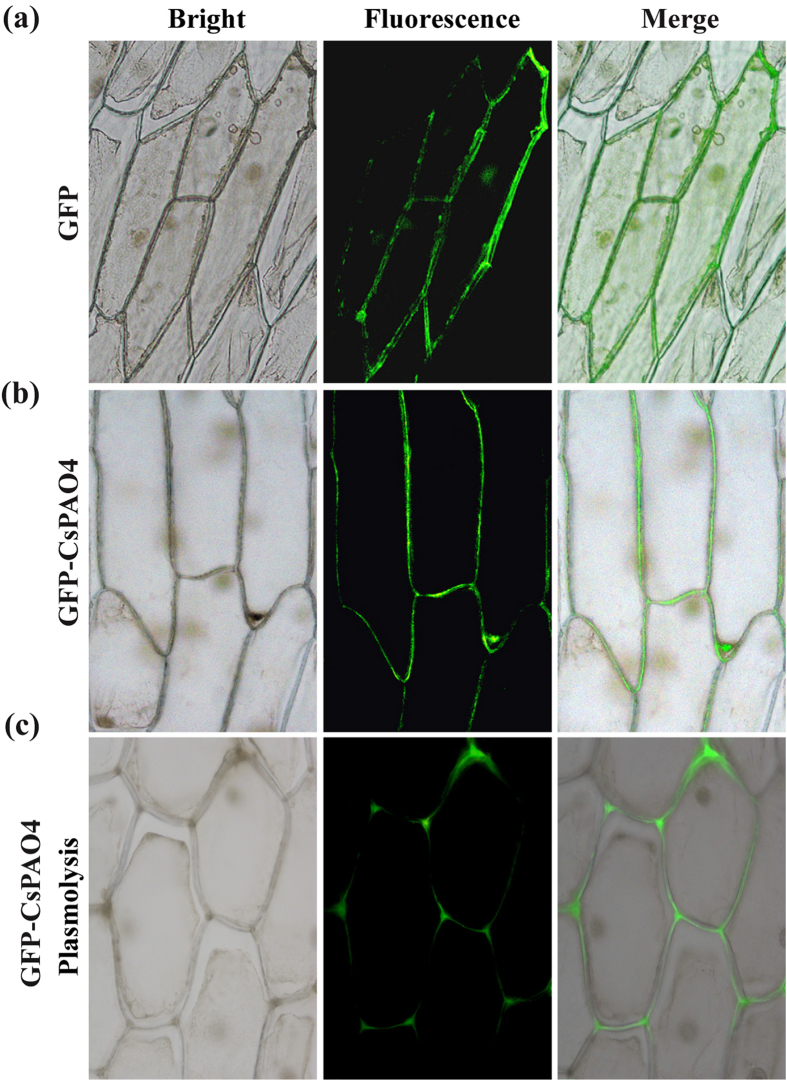
Subcellular localization of CsPAO4. (**a**) Fluorescence signals derived from GFP. (**b**) Fluorescence signals derived from GFP-CsPAO4 fusion protein. (**c**) Fluorescence signals derived from GFP-CsPAO4 fusion protein after plasmolysis. The images are taken under bright (left) and fluorescence (middle) field, respectively, and the merged ones are shown on the right.

To further understand the potential function of *CsPAO4*, subcellular localization of CsPAO4 was investigated. To this end, 35S:CsPAO4-GFP vector was constructed and transformed into onion epidermal cells. After observation with fluorescence microscope, the control GFP was observed throughout the cell, whereas *CsPAO4* was revealed to be localized to the periphery of plant cells, including cell wall, plasma membrane and intercellular space (Fig. 1a,b). However, the de-association of CsPAO4-GFP fluorescence from the plasma membrane after plasmolysis allowed plasma membrane to be ruled out, suggesting that CsPAO4 was located in the apoplast (Fig. 1c).

### *CsPAO4* is involved in PAs terminal catabolism

**Figure 2:**
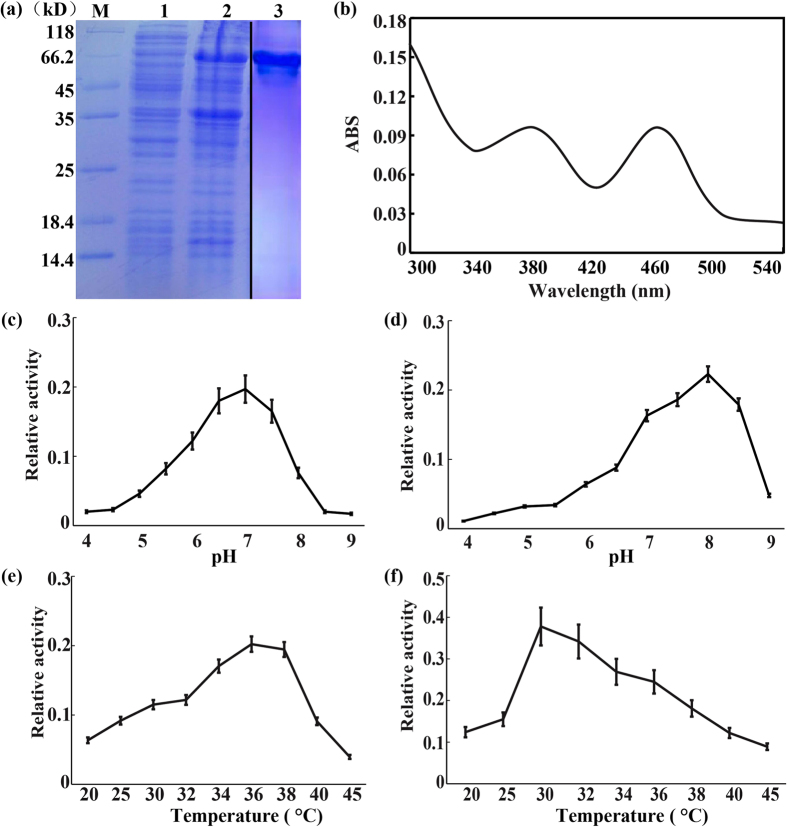
Characterization of recombinant CsPAO4. (**a**) Purification of CsPAO4. M, protein size marker; lane 1, *E. coli* whole-cell lysate without IPTG induction; lane 2, *E. coli* whole-cell lysate with IPTG induction; lane 3, purified CsPAO4. (**b**) Absorbance spectrum of the purified CsPAO4, ranging from 300 to 540 nm. (**c**) Optimum pH for measuring CsPAO4 activity using Spd as a substrate. The buffers used include the following: pH 4.0–5.0, 100 mM MES buffer, pH 5.5–9.0, 100 mM phosphate buffer. (**d**) Optimum pH for measuring CsPAO4 activity using Spm as a substrate. (**e**) Optimum temperature for measuring CsPAO4 activity using Spd as a substrate at pH 7.0. (**f**) Optimum temperature for measuring CsPAO4 activity using Spm as substrate at pH 8.0.

**Figure 3:**
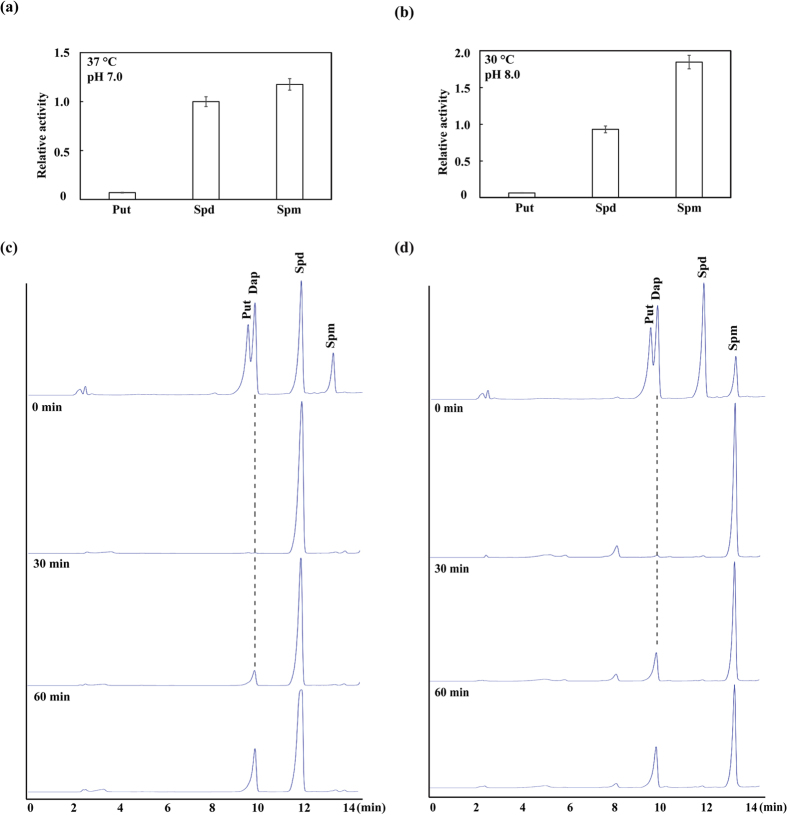
PAs substrate specificity of recombinant CsPAO4 and analysis of the CsPAO4-catalyzed reaction products of Spd and Spm. (**a**) The enzymatic activity of recombinant CsPAO4 was determined in 100 mM phosphate buffer at pH 7.0 and 37 °C. (**b**) The enzymatic activity of recombinant CsPAO4 was determined in 100 mM phosphate buffer at pH 8.0 and 30 °C. (**c,d**) HPLC analysis of reaction products from Spd (**c**) and Spm (**d**), respectively. The top row shows the Put, Dap, Spd and Spm standard; the second and bottom rows show products analysis after incubation with CsPAO4 for 0, 30 and 60 min, respectively.

The recombinant CsPAO4 protein was successfully induced in *E. coli* (Fig. 2a). After extraction and purification, the recombinant CsPAO4 protein exhibited two absorption peak at 380 and 460 nm, indicating a typical feature of FAD-contained protein (Fig. 2b). Two substrates, Spd and Spm, were used to investigate the optimal pH values and temperatures for the highest activities of CsPAO4. The result showed that the optimal pH was pH 7.0 for Spd and pH 8.0 for Spm (Fig. 2c,d), while the optimal temperature was 37 °C for Spd and 30 °C for Spm (Fig. 2e,f). Then substrate specificity of CsPAO4 at pH 7.0, 37 °C and pH 8.0, 30 °C were examined using Put, Spd and Spm. Addition of the three PAs to the CsPAO4 catalytic reaction system showed that Spd and Spm greatly induced the PAO activity, which was not altered by Put (Fig. 3a,b), implying that CsPAO4 used both Spm and Spd as substrates. HPLC analysis showed that when Spd and Spm were incubated in the CsPAO reaction system, a noticeable increase in Dap, concomitant with a decrease of Spd and Spm (Fig. 3c,d), suggesting that CsPAO4 was involved in PAs terminal catabolism reactions.

### Overexpression of *CsPAO4* decreased PA levels, but increased H_2_O_2_

**Figure 4:**
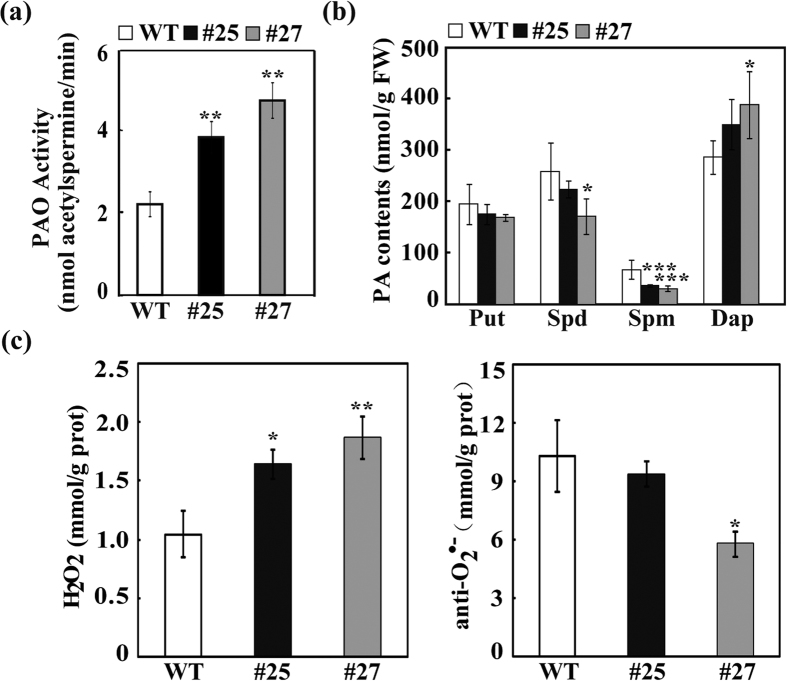
Analysis of PAO activity, free polyamines and ROS levels in wild type (WT) and transgenic plants (#25 and #27). (**a**) PAO activities in the wild type (WT) and transgenic lines (#25 and #27). (**b**) Levels of free putrescine (Put), spermidine (Spd), spermine (Spm) and 1, 3-Diaminopropane (Dap) in WT and transgenic lines. (**c**) H_2_O_2_ and O_2_^•−^ contents in WT and transgenic lines. Error bars represent standard deviations (n = 3). Asterisks indicate significant difference between transgenic lines and WT (*P < 0.05, **P < 0.01, ***P < 0.001).

To investigate the specific function of *CsPAO4*, we generated transgenic tobacco plants overexpressing *CsPAO4* via *Agrobacterium*-mediated transformation (Fig. S2a). Two transgenic lines (#25 and #27) with high expression level of *CsPAO4* were selected for further analysis (Fig. S2b,c). PAO enzyme activity was assessed in both transgenic lines and WT plants under normal growth conditions, which showed that the PAO enzyme activity was significantly elevated in transgenic lines compared to WT plants (Fig. 4a). The free PAs and Dap levels were also analyzed; both Spd and Spm were prominently and significantly decreased, especially for Spm in the transgenic plants in comparison with those in the WT. Meanwhile, Put level in the transgenic lines was also slightly decreased when compared with the WT. By contrast, Dap, one of the products derived from Spd and Spm catabolism, was obviously increased in the transgenic lines compared to WT plants (Fig. 4b).

**Figure 5:**
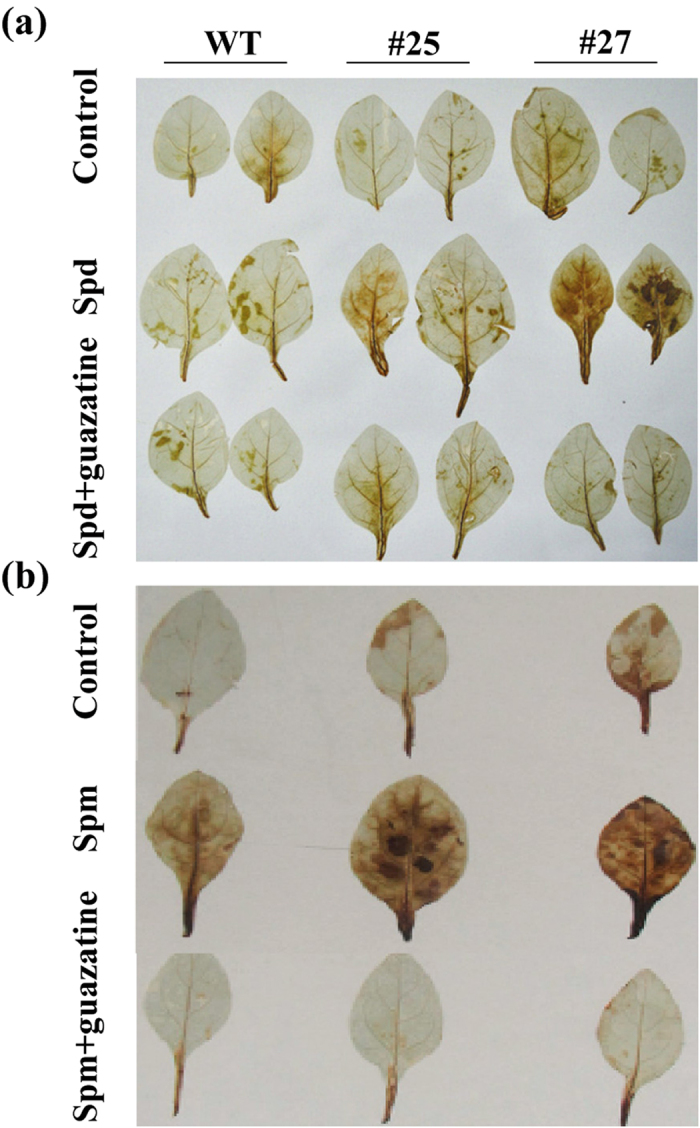
Analysis of H_2_O_2_ in wild type (WT) and transgenic lines (#25 and #27), as revealed by histochemical staining with diaminobenzidine (DAB). (**a,b**) *In situ* accumulation of H_2_O_2_ in leaves treated without (Control, upper panel) or with 1.0 mM Spd (**a**) or Spm (**b**) (middle panel) and 1.0 mM Spd or Spm + 20 μM guazatine (bottom panel).

Since PAO catalyzes polyamine catabolism and leads to H_2_O_2_ production, we are interesting to investigate the ROS content in transgenic lines. Under normal growing conditions, the level of H_2_O_2_ was significantly increased in the transgenic lines when compared with that of WT (Fig. 4c). Surprisingly, the level of O_2_^•−^ was also markedly increased in the transgenic lines. To further verify whether the increased H_2_O_2_ was produced due to oxidation of PAs through PAO, exogenous PAs were supplied and *in situ* H_2_O_2_ accumulation was detected. After treatment with 1 mM Spd or Spm, H_2_O_2_ was significantly increased in transgenic lines, as shown by deeper staining of the leaves, compare to WT. This is particularly obvious in the case of application of exogenous Spm. By contrast, when the transgenic plants were supplied with 20 μM guazatine, a PAO specific inhibitor, H_2_O_2_ was dramatically decreased (Fig. 5a,b). All of these results indicate that the PAO-mediated PAs catabolism accounted for the production of H_2_O_2_ in the transgenic plants.

### Seed germination is promoted in the transgenic lines under salt stress

**Figure 6:**
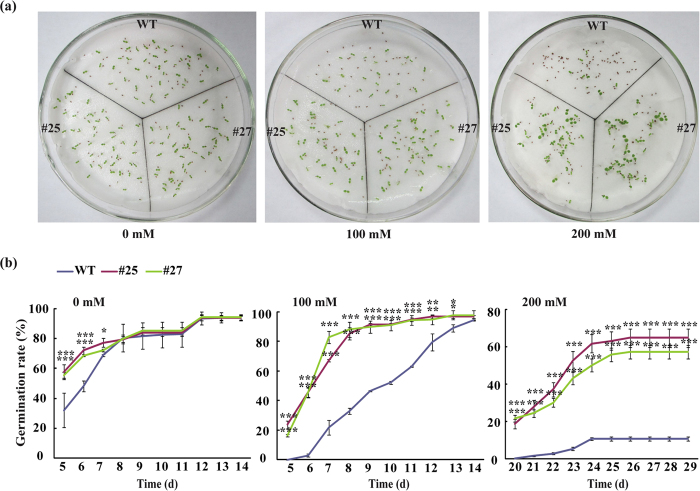
Seed germination of wild type (WT) and transgenic lines (#25 and #27) under salt stress. (**a,b**) Seed germination phenotype (**a**) or time-course change in seed germination rates (**b**) of WT and transgenic lines on MS medium supplemented with 0, 100 and 200 mM NaCl for 30 d. Error bars represent standard deviations for three plates. Asterisks indicate significant difference between WT and transgenic lines at the same time point (*P < 0.05, **P < 0.01, ***P < 0.001).

Salt stress tolerance of the transgenic lines was assessed. First, seed germination on MS basic medium supplied with different concentrations of NaCl was investigated. Under normal conditions (MS without salt), the transgenic seeds germinated in a quicker manner than the WT within the first week, but the difference was not observed from the 8^th^ d afterwards until the end of experiment (Fig. 6a). Significantly higher seed germination rate was detected in the transgenic lines compared to the WT in the presence of 100 mM NaCl, with the exception of last time point when the germination rate was similar between WT and transgenic lines. In the presence of 200 mM NaCl, the seed germination of WT and transgenic seeds was markedly inhibited; however, it is noticeable that the transgenic lines displayed significantly higher germination rate relative to the WT at each time point (Fig. 6a,b). At the last day, the germination rates of #25 and #27 were 64% and 57%, respectively, whereas the WT had a germination rate of 10%. The seeds of both WT and transgenic lines did not germinate when they were grown on MS medium supplemented with 300 mM NaCl, implying that this salt concentration may be lethal to seed germination (data not shown). These data suggested that overexpression of *CsPAO4* led to enhanced salt tolerance of the transgenic seeds. Meanwhile, we also detected the expression of *CsPAO4* under hormone treatment, including ABA and GA_3_. The result showed that the expression of *CsPAO4* was down-regulated by both ABA and GA_3_ (Fig. S4).

### Overexpression of *CsPAO4* inhibits plant growth under salt stress and Spm treatment

**Figure 7:**
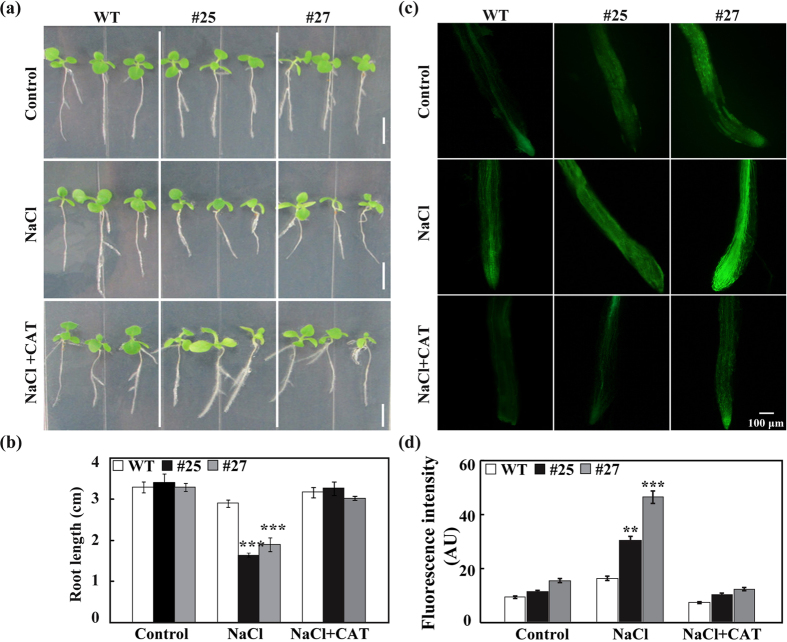
Root growth and H_2_O_2_ accumulation of wild type (WT) and transgenic lines (#25 and #27) under salt stress. (**a,b**) Phenotype (**a**) and quantitative root length (**b**) of four-day-old seedlings of WT and transgenic plants grown on MS medium (Control, upper panel), MS + 100 mM NaCl (middle panel) and MS + 100 mM NaCl + 100 U/ml CAT (bottom panel). (**c**) Fluorescence of primary root tips from six-day-old seedlings of WT and transgenic lines grown in water (Control, upper panel), 200 mM NaCl (middle panel) and 200 mM NaCl + 100 U/ml CAT (bottom panel) for 4 h. (**d**) Quantitative analysis of fluorescence intensity in (**c**). AU, arbitrary units. Error bars represent standard deviations (n = 3). Asterisks indicate significant difference between WT and transgenic lines (*P < 0.05, **P < 0.01, ***P < 0.001).

Then the growth of WT and transgenic plants under salt stress was investigated. First, root growth of WT and transgenic plants under salt stress was observed. When grown on MS medium without salt no difference in root length was detected between WT and transgenic lines (Fig. 7a,b). However, in the presence of 100 mM NaCl, root growth of the transgenic plants was markedly impaired compared to WT, as shown by >40% of root length decrease relative to 10% in WT. When CAT, a H_2_O_2_ scavenger, was added along with 100 mM NaCl to MS medium, the root growth of transgenic lines was restored, and the growth was comparable to the level under control conditions. By contrast, when 20 μM guazatine was added, the root growth inhibition was not restored, but slightly mitigated in comparison with the growth observed without the inhibitor treatment (data not shown). The H_2_O_2_ content in the root tips under salt stress was also examined using the specific dye under fluorescence microscopy. In the absence of salt stress, green fluorescence was comparable in WT and the transgenic plants. When 4-d-cold seedlings were exposed to 200 mM NaCl for 4 h, both WT and transgenic plants displayed enhanced fluorescence in comparison with the normal growth conditions, whereas the fluorescence intensity in the transgenic seedlings was much greater than in the WT. When CAT was added to the seedlings treated with NaCl, the H_2_O_2_-associated fluorescence of both WT and transgenic plants was substantially decreased, but the transgenic lines still showed stronger fluorescence intensity (Fig. 7c,d).

**Figure 8:**
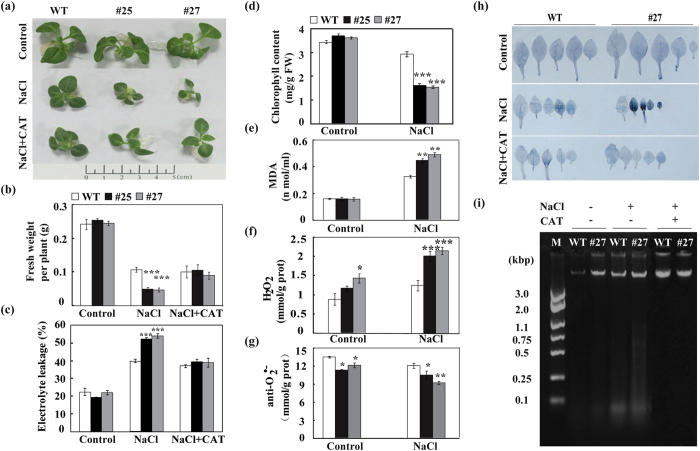
Growth of wild type (WT) and transgenic plants (#25 and #27) under salt stress treatment. (**a–c**) Phenotype (**a**), fresh weight (**b**) and electrolyte leakage (**c**) of WT and transgenic plants grown for 30 days on MS medium (Control, upper panel), MS medium added with 200 mM NaCl (middle panel) or MS medium added with 200 mM NaCl and 100 U/ml CAT (bottom panel). (**d–g**) Chlorophyll content (**d**), MDA content (**e**), H_2_O_2_ (**f**) and O_2_^•−^ (**g**) in WT and transgenic plants under control and salt stress conditions. (**h–i)** Analysis of cell death (**h**) and DNA laddering profiles (**i**) of the leaves sampled from WT and transgenic plants grown on MS (control), MS + 200 mM NaCl or MS + 200 mM NaCl + 100 U/ml CAT. Error bars represent standard deviations for three replicates. Asterisks indicate significant difference between WT and transgenic lines under the same growth conditions (*P < 0.05, **P < 0.01, ***P < 0.001).

Aerial parts of transgenic plants and WT under long-term salt stress were also measured. Under normal growing conditions, the transgenic lines were morphologically indistinguishable from those of WT. When grown on MS medium supplemented with 200 mM NaCl, the growth of aerial parts in WT and the transgenic plants was obviously inhibited, as shown by the reduction of leaf size. However, growth inhibition of the transgenic plants was greater, as manifested by significantly lower fresh weight (FW) of the aerial parts in these plants compare to the WT (Fig. 8a). Under the salt stress FW of the transgenic plants was decreased by more than 80%, while WT exhibited a decrease of 55% (Fig. 8b). When exogenous CAT was applied along with the salt stress, growth of the transgenic plants was largely restored to that of WT. The electrolyte leakage and the contents of chlorophyll and MDA, three important parameters associated with salt stress, were also detected in the WT and transgenic plants. Under normal growth conditions, no significant difference in these parameters was observed among the transgenic plants and WT. Under salt stress, however, the transgenic lines exhibited markedly lower chlorophyll content but higher electrolyte leakage and MDA content, compared to WT (Fig. 8c–e).

As PAOs catalyze the catabolism of Spd and Spm, attempts were made to investigate the root growth of transgenic plants and WT on MS medium or MS medium containing 0.5 mM Spm. There was no dramatic difference in plant growth among the examined genotypes when they were grown on MS medium. When 0.5 mM Spm added to the MS medium, the growth of transgenic lines was significantly inhibited in comparison with the WT (Fig. S5a), and the inhibition was mainly detected in the roots, but not in the aerial parts. The transgenic lines showed 30% lower root length than the WT after 10-d culture on the Spm-containing medium (Fig. S5b). It is worth mentioning that Spm supply in the medium did not affect the root growth of WT when compared with that without Spm treatment. In order to investigate whether Spm-mediated root growth in the transgenic lines was associated with ROS, H_2_O_2_ scavenger CAT was added along with Spm. The inhibition of root growth in transgenic plants under Spm treatment was greatly alleviated by CAT, as the root length was only negligibly smaller than or equivalent to that of WT (Fig. S5a,b). We also analyzed H_2_O_2_ accumulation in the primary roots of WT and transgenic lines before and after Spm treatment. Without the Spm treatment, fluorescence intensity in the root of transgenic line was slightly stronger than in WT. When exogenous Spm was applied, the fluorescence in both WT and transgenic plant was obviously elevated, but the change was greater in the root of transgenic line. When CAT was added along with Spm, the H_2_O_2_-mediated fluorescence was profoundly decreased compared to Spm treatment alone (Fig. S5c). It has to be pointed out that fluorescence intensity of the transgenic line was still greater than that of WT. These findings suggested that the inhibitory effect of Spm on the growth of *PAO*-overexpressing plants was largely ascribed to overproduction of H_2_O_2_.

### PCD was induced in the transgenic lines under salt stress

Efforts were made to investigate whether growth inhibition of the *CsPAO4*-overexpressing transgenic lines under salt stress was associated with ROS accumulation. For this purpose, the ROS levels in the WT and transgenic plants after salt stress were measured. H_2_O_2_ and O_2_^•−^ in the transgenic lines were obviously increased compared to those of WT when they were grown on MS medium supplemented with 200 mM NaCl (Fig. 8f,g). Meanwhile, the antioxidant enzyme activity was also investigated. Under normal growing conditions, activities of POD, CAT and SOD were significantly increased in transgenic lines compared to that of in WT which was consistent with the increased ROS content. On the contrary, under salt stress, activities of these antioxidant enzymes were obviously increased in WT, whereas it was only slightly increased or even decreased in the transgenic lines (Fig. S6a–c). Furthermore, we analyzed the expression levels of some stress-related genes in WT and transgenic lines, such as antioxidant genes (*NtSOD*, *NtCAT* and *NtPOX2*), ABA synthetic gene (*NtNCED3*), late embryogenesis abundant protein gene (*NtLEA5*) and salt stress response gene (*NtSOS1*). Under control conditions, the expression levels of these genes in WT were much higher than in the transgenic lines (Fig. S7). However, under saline conditions most of these genes were down-regulated in both WT and transgenic lines except *NtSOS1*. The transcript levels of these genes in the transgenic lines are much higher than those of the WT (Fig. S7). The expression level of *NtSOS1* was obviously increased in both WT and transgenic plants under salt stress treatment, especially in the transgenic lines. As ROS is a key factor leading to PCD, PCD pattern of the WT and transgenic lines was assessed based on trypan blue staining and DNA fragmentation. No obvious staining was observed in both WT and transgenic lines under control conditions (Fig. 8h). Upon exposure to the salt stress, the staining was much deeper in both WT plants and transgenic lines compared to the control conditions. However, staining in the transgenic lines was stronger than in the WT, indicating that the transgenic lines displayed more serious PCD. When CAT was used along with the salt, staining of transgenic lines was equivalent to the WT, and both of them were recovered to the control level (Fig. 8h). DNA fragmentation was examined by agarose gel electrophoresis using total DNA derived from the seedlings treated with salt stress. No obvious DNA laddering was found in either WT or transgenic lines under control conditions. Salt stress led to DNA laddering in both WT and the transgenic lines, but more serious pattern was detected in the latter, implying that the transgenic lines suffered from more severe DNA fragmentation or PCD (Fig. 8i). By contrast, when CAT was supplied in the salt medium, DNA laddering was greatly alleviated. Collectively, these results suggest that PCD in the transgenic lines largely resulted from the H_2_O_2_ produced under salt stress.

### Oxidation of Spd and Spm was promoted in the transgenic lines

In order to know whether the PAs oxidation differed between the transgenic lines and WT, we analyzed PAO activity and the PAs levels in the plants grown with or without salt stress. As shown in Fig. S3a, PAO activities in the two transgenic lines were significantly higher than in the WT before and after the salt stress. Consistent with the increased PAO activity in the transgenic lines, Spd and Spm contents in these lines were dramatically decreased compared to WT. On the contrary, level of Dap was prominently increased in the transgenic lines as compared to the WT (Fig. S3b). Under salt stress, contents of PAs and Dap were increased in both WT and transgenic lines compared to unstressful conditions. It is noticeable that Spd and Spm contents in the WT were still significantly higher than in transgenic lines. Similar to the control conditions, prominently higher level of Dap content was detected in the transgenic lines in comparison with the WT (Fig. S3c). These results demonstrated that Spd and Spm oxidation was promoted in the transgenic lines, consistent with the higher PAO activities. No significant difference in Put level was observed among the tested lines before salt stress, but the putrescine level of WT was slightly higher than those of transgenic lines after imposition of salt stress.

## Discussion

It has been well documented that PA catabolism plays important roles in plant development and stress response. PAs catabolism is catalyzed by PAOs that are involved in either terminal catabolism or back-conversion reactions depending on the end products; these two groups of PAOs have been demonstrated to exist in different plant species up to now. However, previous studies primarily focus on PAOs of model plants or herbaceous plants, such as *Arabidopsis* and rice, but information on PAOs of perennial woody plants is quite limited. On the other hand, most of the identified PAOs are predominantly supposed to be implicated in back conversion reactions of PAs catabolism, whereas those pertinent to terminal degradation are less well characterized. In this study, we cloned a PAO gene *CsPAO4* from sweet orange and further investigated its function in PAs catabolism and salt stress response. Amino acid sequences alignment showed that CsPAO4 was different from other PAO genes implicated in PA terminal catabolism, as it lacks the signal peptide (SP) that is crucial for protein localization^20^. So far, maize PAO (ZmPAO), rice PAO (OsPAO7) and barley PAO (HvPAO1) with the SP have been proved to be localized in apoplast^20^,^34^. However, another barley PAO (HvPAO2) with the SP is a symplastic protein^34^. It has been reported that proteins without SP can also be secreted by unconventional mechanism, which is termed unconventional protein secretion (UPS)^35^. In plants even 50% of secreted proteins can be UPS dependent^36^. Cellular localization of PAO protein is a critical criterion for distinguishing their roles in terminal catabolism or back-conversion reactions. Several earlier studies suggest that the PAOs involved in PAs terminal catabolism are localized in apoplast, while PAOs associated with PAs back conversion are located in intracellular space^20^,^25^,^26^. In this study, we found that CsPAO4 was localized in apoplast, providing an evidence for supporting its involvement in PAs terminal catabolism. Meanwhile, CsPAO4 is probably a UPS-dependent secreted protein. We further determined the role of CsPAO4 in terminal catabolism based on detecting reaction products by supplying substrates to this protein, as done elsewhere^25^,^26^,^27^, which provides convincing evidence supporting the implication of CsPAO4 in PAs terminal catabolism. On the other hand, we also analyzed the PAs levels and one of the major catabolic products, Dap, in the transgenic plants overexpressing *CsPAO4*. Spd and Spm were decreased in the two transgenic lines, concurrent with elevation of Dap, compared to those of WT, which is consistent with the terminal catabolism of Spd and Spm. Surprisingly, putrescine levels in the transgenic lines were also slightly decreased relative to the WT. However, this result is not unique, as Moschou *et al.*^37^ has also reported that overexpression of a maize PAO gene, *Zmpao*, in tobacco led to reduction of putrescine level in the transgenic plants. One explanation is that the PAO-mediated Spd and Spm catabolism caused a feedback on the activation of synthesis of these two compounds from the first precursor in the PAs pathway.

Current researches support that PAOs of monocots catalyze PAs terminal catabolism, while PAOs of dicots are involved in PAs back-conversion reactions^17^,^21^. So far, the PAOs in *Arabidopsis* and rice have been well studied, and their roles in either terminal catabolism or back conversion are elucidated. Up to now, all of the PAO members in *Arabidopsis* PAO family are proposed to catalyze the PAs back conversion reactions^21^,^22^,^23^,^24^,^25^, whereas the rice PAOs are involved in both catabolic pathways^20^,^26^,^27^. Recently, we have reported that a sweet orange PAO, CsPAO3, is likely to catalyze PAs back conversion^29^. Different from CsPAO3, CsPAO4, another sweet orange PAO member in the same family, catalyzes PAs terminal catabolism, suggesting that PAO members of citrus might function in either PAs terminal catabolism or back conversion, which is similar to the situations of rice. CsPAO4 represents the first PAO of dicots that participates in PAs terminal catabolism. These findings demonstrate that the PAOs from the same plant species display functional diversity and that they may function in different manners. On the other hand, it is possible that PAs catabolism in citrus and rice may exhibit a more complex pattern than in *Arabidopsis*, but this hypothesis needs to be verified in the future.

It has been well documented that H_2_O_2_ derived from PAO-mediated PAs oxidation takes part in many physiological processes in higher plants, including development, biotic and abiotic stresses response^38^,^39^,^40^. In our work, *CsPAO4* was overexpressed in the transgenic plants, which had higher PAO activity and increased levels of H_2_O_2_ in comparison with the WT under normal conditions. On the other hand, the transgenic lines displayed enhanced accumulation of H_2_O_2_ compared to the WT when they were treated with exogenous Spd and Spm, but the H_2_O_2_ accumulation was indistinguishable from each other when PAO inhibitor was used along with Spd and Spm. All of these results indicate that PAO-mediated PA catabolism accounted for the increased amount of H_2_O_2_ in the transgenic plants. In this study, we found that the transgenic plants were more sensitive to salt stress compared to the WT at seedling stage, as manifested by shorter root, reduced fresh weight, decreased chlorophyll content and increased electrolyte leakage and MDA content. This result is in agreement with an earlier study by Moschou *et al.*^22^ who reported that overexpression of a *PAO* gene led to repression of vegetative growth under salt stress. One explanation for this phenomenon is that under salt stress Spd or Spm was increasingly accumulated and then secreted into apoplast where they are oxidized by PAO to generate abundant amount of H_2_O_2_. It is known that H_2_O_2_ plays a dual role in plant stress response; at low concentration, H_2_O_2_ acts as a signaling molecule to trigger the expression of stress-responsive genes to fight against various environmental stresses, whereas at high concentration it causes oxidative stress and induces PCD^41^,^42^. It is conceivable that under salt stress H_2_O_2_ in the *CsPAO4*-overexpressing plants may accumulate to a threshold value, beyond which it acts as a toxic compound to induce either oxidative stress or PCD. We thus speculate that the PAO-derived H_2_O_2_ was associated with the observed salt sensitive response in the transgenic lines. This speculation is supported by the following findings. First, salt susceptibility of transgenic plants is concurrent with higher level of H_2_O_2_ relative to WT under salt stress. Second, H_2_O_2_ scavenger application dramatically decreased H_2_O_2_ level in the transgenic plants and resumed the root growth under salt stress, whereas PAO inhibitor application partly abolished the negative effect of salt stress on root growth. Third, PCD in the transgenic plants was stimulated under salt stress, whereas application of CAT disrupted PCD. Lastly, exogenous supply of Spm mimicked the salt stress-derived root growth retardation, which was resumed to normal situations in the presence of CAT.

However, it is worth mentioning that growth inhibition of the transgenic seedlings was possibly caused by the disruption of a dynamic balance between PA anabolism and catabolism due to the overexpression of a *PAO* gene in the transgenic plants. It has been suggested that a high ratio of PA anabolism to catabolism may result in PA accumulation, while a low ratio suggests that more PAs are used for PAO-mediated oxidization^22^. In general, the balance between PA biosynthesis and catabolism is tightly and delicately regulated in plants. However, overexpression of *CsPAO4* might break this balance, and PA catabolism predominated over PA anabolism in the transgenic lines. As a consequence, they accumulated less PAs in the presence of salt stress in comparison with the WT, which is largely true based on the analysis of endogenous PA levels. PAs have been suggested to act as important stress molecules and function to promote plant tolerance to various abiotic stresses through stabilizing membranes and promoting ROS scavenging^43^,^44^,^45^. Therefore, it is assumed that the salt stress susceptibility is partly ascribed to the lower accumulation of PAs in the transgenic plants, which possibly failed to overcome the salt-derived cell damages due to impotent capacity for mitigating the stresses.

Although overexpression of *CsPAO4* led to salt susceptibility at the vegetative growth stage, we found that the transgenic plants exhibited better salt tolerance than WT at germination stage, as shown by faster germination time and higher germination rate. It suggests that plants responses to a given stressor may vary depending on the developmental stages, which has been also documented in earlier studies^46^. However, our result is different from that of Moschou *et al.*^14^ who found that seed germination of transgenic plants overexpressing a *PAO* gene was impaired in the presence of salt stress. The underlying reason for these contrasting results in different studies remains unclear. Based on our data it is assumed that the overproduction of H_2_O_2_ contributes to the accelerated seed germination under salt stress. H_2_O_2_ has been demonstrated to act as a priming factor to play a key role in coordination of seed germination^47^,^48^,^49^. In another study, Çavusoglu and Kabar^50^ also proved that H_2_O_2_ was useful to overcome germination delay and functions to prevent salt stress-derived negative effects. These findings, together with ours, point to the positive role of H_2_O_2_ in promoting seed germination under abiotic stress. Seed germination is a highly complex process that is regulated by an array of endogenous and exogenous factors, of which ABA has been shown to act as a major signaling phytohormone. ABA has been shown to inhibit seed germination of different plants^51^,^52^. ABA signaling cascade involves a number of intermediate molecules, including H_2_O_2_, to relay the relevant signals to downstream targets. ABA is also verified to act as a key signal to induce oxidation of PAs and regulates the generation of H_2_O_2_ under environmental stress^53^. Therefore, it is assumed that overproduction of H_2_O_2_ in the transgenic plants overexpressing *CsPAO4* may have a feedback inhibitory effect on the ABA signaling associated with inhibition of seed germination. Therefore, high level of H_2_O_2_ in transgenic lines might reverse the inhibitory effect of ABA on seed germination under salt stress.

Taken together, in this study we functionally characterized CsPAO4 of sweet orange. Transgenic approach demonstrates that *CsPAO4* functions in polyamine terminal catabolism to produce H_2_O_2_. Overproduction of H_2_O_2_ in the transgenic plants has been shown to be associated with the contrasting tolerance to salt stress at germination and seedling stage. To our knowledge, this is first report on elucidating a *PAO* gene involved in terminal catabolism from a perennial plant, and the finding from this study provides knowledge for modulating salt stress tolerance via genetic manipulation of a PAO gene.

## Materials and Methods

### Plant materials and treatments

Six-year-old sweet orange plants grown in greenhouse were used to examine the gene expression patterns under different treatments. Uniform and healthy shoots were selected from the plants and inserted in a flask containing distilled water, which were kept for 1 d in a growth chamber at 26 °C with 16-h light/8-h dark photoperiod. For hormone treatment, the shoots were transfered into distilled water containing 100 μM ABA or 100 mg/L GA_3_, respectively, and cultured under above conditions. The samples were collected at 0, 6, 12, 24, 48 and 72 h after treatment. All the samples were frozen immediately in liquid nitrogen and stored at −80 °C until using for RNA extraction.

### Quantitative real-time RT-PCR (qPCR) analysis

Total RNA was extracted with RNAiso Plus (TaKaRa, Dalian, China) and treated with DNase I (TaKaRa, Dalian, China) according to the manufacturer’s recommendations. The quality of total RNA was detected with a spectrophotometer (NanoDrop ND-2000, Wilmington, DE, USA). About 1 μg of total RNA was used to synthesize first-strand cDNA with PrimeScript^®^ First Strand cDNA Synthesis Kit (TaKaRa, Dalian, China) according to the manufacturer’s instructions. The primers used for qPCR were listed in table S1. The qPCR reaction solutions included 5 μL of SYBR Green PCR master mix (QIAGEN, Germany), 1 μL of diluted cDNA, 0.25 μL of each primer and 3.5 μL of RNase-free H_2_O. qPCR was performed on a QuantStudio 7 Flex system (Applied Biosystems, USA) with the following cycling conditions: 50 °C for 2 min, 95 °C for 5 min and 40 cycles of 95 °C for 10 s, 56–63 °C (depending on the primers) for 30 s and 72 °C for 20 s. The expression level of each sample was calculated with 2^−∆∆CT^ method (Livak and Schmittgen)^54^ after normalization with citrus *β*-actin gene, which was used as an internal control. Four replicates were used for each sample. The relative expression levels were log2 transformed to generate heat map representation using Cluster 3.0 software.

### Gene isolation and sequence analysis

Six putative polyamine oxidase genes were previously identified in sweet orange genome^29^, of which *CsPAO4* was PCR amplified with primers ACsPAO4_F/R_ (Table S1, in which the primers of this study are listed, unless otherwise stated). The PCR product was inserted in the vector pMD18-T, transformed into *E. coli* and sequenced. Multiple sequence alignments between *CsPAO4* and other PAO genes involved in PAs terminal catabolism were performed by ClustaX (version 1.81) and Genedoc (version 2.7).

### Subcellular localization analysis

The *CsPAO4* ORF was amplified using the primer LCsPAO4 _F/R_ with the *Stu*I and *Mlu*I restriction sites, and ligated to the N terminal of a GFP gene in the pCAMBIA1302 vector that was linearized with *Stu*I and *Mlu*I, yielding 35S::CsPAO4-GFP. The resulting construct was transferred into onion epidermal cells by *Agrobacterium tumefaciens* (strain GV3101)-mediated infection. The control construct (pCAMBIA1302 alone) was used as control. After infection, the onion epidermal cells were cultured on 1/2 MS basal medium at 26 °C in dark for 72 h and then observed with a fluorescence microscope (ECLIPSE 90i; Nikon, Japan). For plasmolysis, the onion epidermal cells were treated with 0.5 M mannitol for 10 min.

### Preparation of recombinant CsPAO4 protein in Escherichia coli

The *CsPAO4* ORF was amplified using the primer YCsPAO4_F/R_ with the *Sal*I and *Pst*I restriction sites, and cloned into the 6 × His tag of the pCold-I vector (TAKARA BIO INC) resulting in pCold-CsPAO4. After confirming the cloned fragments by DNA sequencing, the pCold-CsPAO4 was transformed into *E. coli* (BL21) cells and recombinant CsPAO4 protein tagged with 6 × His was produced according to the manufacturer’s instructions (TAKARA BIO INC). The transformant was inoculated in the medium including 100 μg/ml of ampicillin and cultured at 37 °C with shaking. At OD_600_ 0.4–0.5 the culture solution was refrigerated at 15 °C and kept still for 30 min. Then IPTG was added to the final concentration of 0.1–1.0 mM, and the culture was shaken gently for 24 h at 15 °C. Subsequently, the cells were collected to confirm the expression of target protein in soluble and insoluble fractions with SDS-PAGE. Protein purification was then performed according to the Ni-NTA SefinoseKit instructions (C600332-0001, BBI, China).

### CsPAO4 protein activity assay

The catalytic activities of recombinant CsPAO4 protein for the oxidation of Put, Spd and Spm were determined as described by Tavladoraki *et al.* (2006)^13^. In order to determine the optimum pH, 100 mM MES buffer for the pH 4.0–5.0 range and 100 mM phosphate buffer for pH 5.5–9.0 range were used. To investigate the optimum temperature, the temperature from 25 °C to 45 °C was determined with the optimum pH have been confirmed above. In a typical experiment, about 5 μg of protein was added to a buffered solution (100 mM phosphate buffer, pH 7.0) containing the substrate (500 μM), 4-aminoantipyrine (100 μM), 3,5-dichloro-2-hydroxybenzesulfonic acid (1 mM), and horseradish peroxidase (10 U/ml), and the increase in absorbance at 515 nm was monitored.

### HPLC analysis of recombinant CsPAO4-generated PAs products

To determine the reaction products of PAs oxidation, 10 μg of purified CsPAO4 protein was incubated with 150 mM Spd in 100 mM phosphate buffer (pH 7.0) at 37 °C or with 150 mM Spm in 100 mM phosphate buffer (pH 7.0) at 30 °C for different time points. The reaction products were extracted by 5% (v/v) perchloric acid (PCA) as described by Flores and Galston^55^. The derivation and benzoylation of the extracted PAs and Dap were performed with the method described by Fu *et al.*^56^. The benzoylated PAs were separated and quantified at ambient environment by HPLC (Waters, Milford MA, US) with a reverse-phase C_18_ column and an UV detector (230 nm). The programmed methanol-water step gradient was applied with a flow rate of 0.7 ml/min (55% methanol and 45% H_2_O at 0 min, 95% methanol and 5% H_2_O at 10 min, 100% methanol at 13 min, 55% methanol and 45% H_2_O at 14 min, 55% methanol and 45% H_2_O at 20 min).

### Vector construction and plant transformation

The full length of cDNA (1710 bp) encoding *CsPAO4* was amplified with primers CCsPAO4_F/R_ including *Sma*I and *Sac*I restriction sites. The sequenced PCR product was fused with the binary pBI121 expression vector under the control of the 35S cauliflower mosaic virus (*CaMV35S*) promoter. The pBI121 vector containing *CsPAO4* cDNA was introduced into *A. tumefaciens* strain GV3101. *Agrobacterium*-mediated leaf disc transformation was used to generate the stable transgenic tobacco^57^. Transformants were selected against 100 mg/L kanamycin on MS medium^58^ and confirmed by PCR with primers SCsPAO4_F/R_ including forward *CaMV35S* promoter primer and reverse gene specific primer for *CsPAO4*. Semi-quantitative RT-PCR method was used to analyze the expression level of *CsPAO4* in transgenic lines with the primers QCsPAO4_F/R_. *Ubiqutin* gene was used as an internal control using specific primers. Two transgenic lines (#25 and #27) of T_2_ generation with high expression levels of *CsPAO4* were selected for further study. Three-week-old seedlings of wide type (WT) and transgenic lines under normal growth conditions were selected for PAO protein activity measurement, PAs analysis and ROS detection.

### Salt stress treatment and growth detection

For salt stress treatment, transgenic lines of T_2_ generation and wild type (WT) plants were used. For germination rate analysis, WT and transgenic seeds were surface-sterilized and sown on MS medium with 0, 100, 200 and 300 mM NaCl. For root elongation assessment under salt stress, the 4-d-old seedlings of uniform size were grown on MS medium containing NaCl (0, 100 and 200 mM) or Spm (0, and 0.5 mM). In another set of work, 4-d-old seedlings were grown on MS medium containing 20 μM guazatine or 100 U/ml catalase (CAT), respectively before they were treated with 100 mM NaCl. Root length was measured after 10 d of treatment on the relevant media. For vegetative growth observation under salt stress, 4-d-old seedlings of uniform size were grown on MS medium containing NaCl (0 and 200 mM). Samples of WT and transgenic plants were collected after 30 d of treatments for measurement of fresh weight (FW), PAs contents, ROS level and DNA fragmentation.

### Analysis of free PAs levels, Dap contents and PAO activities

The extraction, derivation and benzoylation of free PAs and Dap were performed as described above. The PAO activity was detected in the leaf samples with plant PAO assay kit (GMS50139.5, Genmed Scientifics Inc. USA) according to the manufacturer’s instruction. Three replicates were performed with independent samples. The protein concentration was detected based on coomassie brilliant blue staining as described by Bradford^59^.

### Detection of antioxidant enzyme activities

About 0.1 g of the leaf samples was homogenized in 900 μl of 100 mM phosphate buffer (pH 7.0) on ice, followed by centrifugation at 10000 rpm for 10 min. Subsequently, 10% of the supernatant was used for measurement of activities of antioxidant enzyme, including peroxidase (POD), superoxide dismutase (SOD) and catalase (CAT) was measured with detection kits designed for these enzymes (A084 for POD, A001 for SOD, A007 for CAT, Nanjing Jiancheng Bioengineering Institute, China) according to the manufacture’s introductions, respectively. Three replicates were performed with independent samples. The protein concentration was detected with the method of coomassie brilliant blue staining as described above.

### *In situ* histochemical detection and measurement of ROS levels

*In situ* accumulation of H_2_O_2_ was detected by histochemical staining with 3, 3′-diamino-benzidine (DAB) as described by Shi *et al.*^60^. For this purpose, the leaves were soaked in fresh 1 mg/ml DAB solution (pH 3.8) until brown spots appeared. After staining, the leaves were destained in 75% ethyl alcohol. To quantitatively measure H_2_O_2_ and O_2_^•−^, nearly 0.1 g of the leaf samples was homogenized in 0.9 ml of 100 mM phosphate buffer (pH 7.0) on ice, and then the homogenate was centrifuged at 10000 rpm for 10 min. Subsequently, 10% of the supernatant was used for measurement of H_2_O_2_ and O_2_^•−^ with detection kits (A064 for H_2_O_2_, A052 for O_2_^•−^, Nanjing Jiancheng Bioengineering Institute, China) according to introductions provided by the supplier. The protein concentration was detected as described above.

### H_2_O_2_ detection by fluorescence microscopy

*In situ* localization of H_2_O_2_ was performed using the highly sensitive, cell-permeable probe 2′, 7′-dichlo-rodihydrofluorescein diacetate (DCFH_2_-DA) as described by Fu *et al.*^56^. In brief, 6-d-old seedlings of WT and transgenic lines were transferred to 96-well culture plates and treated with double distilled H_2_O (ddH_2_O, as control), 200 mM NaCl and 200 mM NaCl + 100 U/ml CAT, respectively, at 26 °C in light for 4 h. Then the seedlings were incubated in 50 μM DCFH_2_-DA dissolved in Tris buffer (10 mM Tris, 50 mM KCl, pH 7.2) in darkness on a rotary shaker (40 rpm) for 20 min. After incubation the seedlings were washed with Tris buffer (10 mM Tris, 50 mM KCl, pH 6.1) to remove excessive dye and observed with a fluorescence microscope (ECLIPSE 90i; Nikon, Japan).

### Measurement of electrolyte leakage, chlorophyll and MDA

Electrolyte leakage (EL) was detected according to Liu *et al.*^61^. In brief, the leaf samples were incubated in a tube containing 20 mL of ddH_2_O, while tube contained only 20 mL of ddH_2_O was used as a control. The tubes were shaken slowly for 90 min on a rotary shaker at room temperature. Initial conductivity of the samples (C_s0_) and control (C_c0_) was measured using a conductivity meter (STARTER 3100C, America). Subsequently, the samples were boiled for 10 min, then cooled down to room temperature. The final conductivity (C_s1_ and C_c1_) of the sample and control was measured as described above. EL, represented by the relative conductance, was calculated using the formula: C(%) = (C_s0_–C_c0_)/(C_s1_–C_c1_) × 100. Chlorophyll content was measured as described by Fu *et al.*^62^. For MDA measurement, 0.1 g of the leaf samples were homogenated in 900 μl of 100 mM phosphate buffer (pH 7.0) on ice, followed by centrifugation at 10000 rpm for 10 min. The supernatant was used for measurement of MDA with a MDA detection kit (A003-1, Nanjing Jiancheng Bioengineering Institute, China) according to the manufacture’s instruction.

### Analysis of DNA fragmentation and PCD

Total DNA from tobacco seedlings after exposure to salt treatments was extracted by cetyltrimethyl ammonium bromide (CTAB) method^63^ with a slight change. For DNA analysis, identical amounts of DNA samples (10μg) were separated on 1.0% (w/v) agarose gel. DNA was visualized by staining with ExRed (ZS203-1, ZOMANBIO, China). PCD was examined by trypan blue staining as described by Pogány *et al.*^64^.

### Statistical analysis

Three replicates were performed with independent samples for each treatment, which was repeated at least twice. Statistical differences were analyzed using ANOVA (analysis of variance) based on Duncan’s multiple range test, taking the significance levels at *P* < 0.05 (*), *P* < 0.01 (**), and *P* < 0.001 (***), respectively.

## Additional Information

**How to cite this article**: Wang, W. and Liu, J.-H. *CsPAO4* of *Citrus sinensis* functions in polyamine terminal catabolism and inhibits plant growth under salt stress. *Sci. Rep.* **6**, 31384; doi: 10.1038/srep31384 (2016).

## Supplementary Material

Supplementary Information
