# Supplementary material for: CsPAO4 of Citrus sinensis functions in polyamine terminal catabolism and inhibits plant growth under salt stress
Source: Sci Rep. 2016 Aug 18;6:31384. doi: 10.1038/srep31384 (PMC4989168; doi:10.1038/srep31384)
Supplement: Supplementary Information [file srep31384-s1.pdf]

CsPAO4 of *Citrus sinensis* functions in polyamine terminal catabolism and inhibits plant growth under salt stress

Wei Wang, Ji-Hong Liu[†]

Supplemental Figure S1. Amino acid sequence alignment of CsPAO4 and PAOs from other plants, including maize (ZmPAO1), barley (HvPAO1, HvPAO2), and rice (OsPAO7). Identical and similar residues are shaded in black and gray background, respectively. Black line above indicates the signal peptide and transmembrane domain (SP+TD), and the asterisks indicate the putative residues involved in catalytic activity.

Supplemental Figure S2. Generation and characterization of transgenic plants overexpressing *CsPAO4*. (a) A construct used for production of *CsPAO4*-overexpressing lines. RB, right border; $NOS_{\textit{pro/ter}}$, nopaline synthase gene promoter/terminator; NPTII, neomycin phosphotransferase II; CaMV 35S, Cauliflower mosaic virus 35S promoter; LB, left border. (b) Molecular characterization of transgenic lines by genomic PCR. P, plasmid. **(c)** RT-PCR analysis the expression of *CsPAO4* in WT and transgenic plants.

Supplemental Figure S3. Analysis of PAO activity and polyamine levels in the transgenic lines. (**a-c**) PAO activity (**a**), free polyamine (**b**) and Dap (**c**) of wild type (WT) and transgenic lines (#25 and #27) under control conditions and salt stress treatment. Error bars represent standard deviations for three replicates. Asterisks indicate significant difference between WT and transgenic lines under the same

growth conditions (* $P<0.05$, ** $P<0.01$, *** $P<0.001$).

Supplemental Figure S4. The expression pattern of *CsPAO4* under hormone treatment. (**a**) The expression of *CsPAO4* under 100 μM of ABA treatment. (**b**) The expression of CsPAO4 under 100 mg/L of GA_3 treatment. Error bars represent standard deviations (n=4).

Supplemental Figure S5. Root growth and H_2O_2 accumulation of wild type (WT) and transgenic lines (#25 and #27) under Spm treatment. (**a-b**) Phenotype (**a**) and quantitative measurement of root length (**b**) of four-d-old seedlings of WT and transgenic plants grown on MS medium (Control, upper panel), MS + 0.5 mM Spm (**middle** panel), MS + 0.5 mM Spm + 100 U/ml CAT (bottom panel). (**c**) Fluorescence of primary root tips from 6-d-old seedlings of WT and transgenic lines grown in water (Control, upper panel), 1 mM Spm (middle panel) and 1 mM Spm + 100 U/ml CAT (bottom panel) for 1 h. (**d**) Quantitative analysis of fluorescence intensity in (**c**). AU, arbitrary units. Error bars represent standard deviations (n=3). Asterisks indicate significant difference between WT and transgenic lines under the same treatment (*** $P<0.001$).

Supplemental Figure S6. The antioxidant enzyme activity of wild type (WT) and transgenic plants (#25 and #27) under control conditions and salt stress. (**a-c**) The POD (**a**), CAT (**b**) and SOD (**c**) enzyme activity of wild type (WT) and transgenic plants. Error bars represent standard deviations for three replicates. Asterisks indicate significant difference between WT and transgenic lines under the same growth conditions (* $P<0.05$, ** $P<0.01$).

Supplemental Figure S7. The expression levels of some stress-related genes in wild type (WT) and transgenic plants (OE) under control conditions and salt stress. Error bars **represent** standard deviations (n=4).

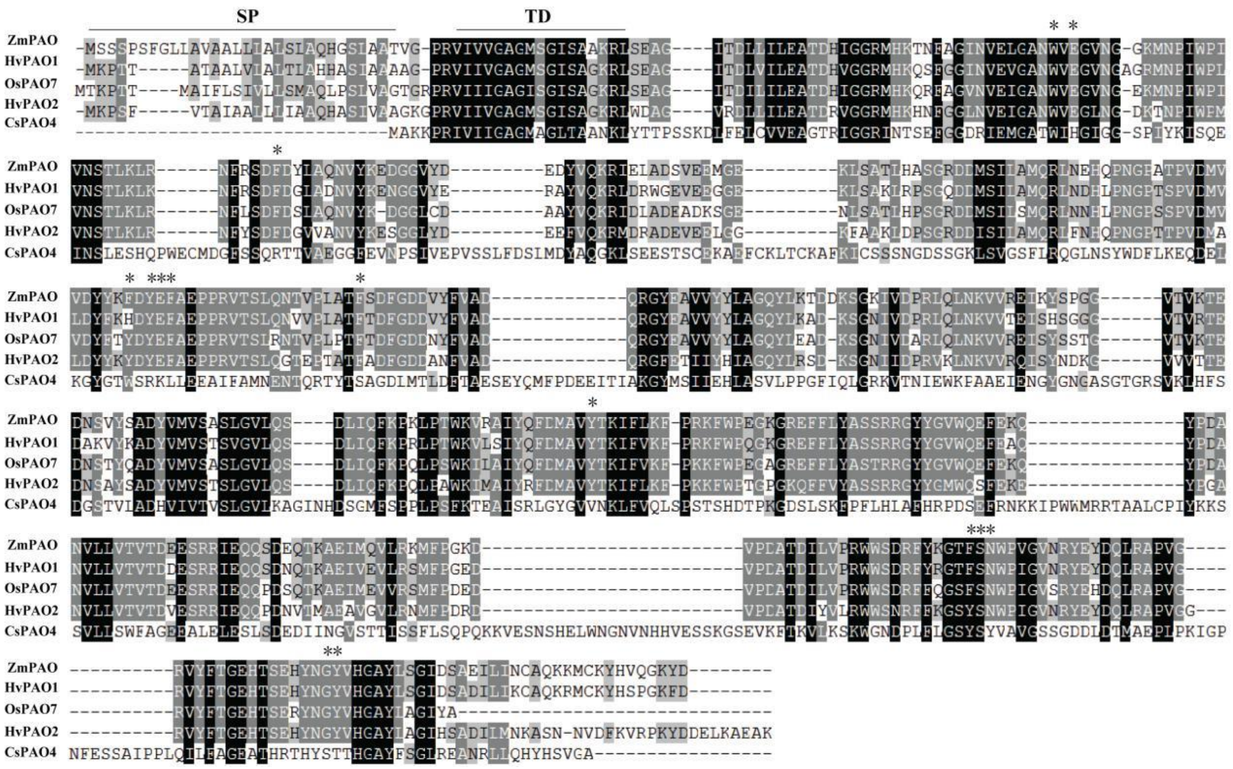

Figure S1

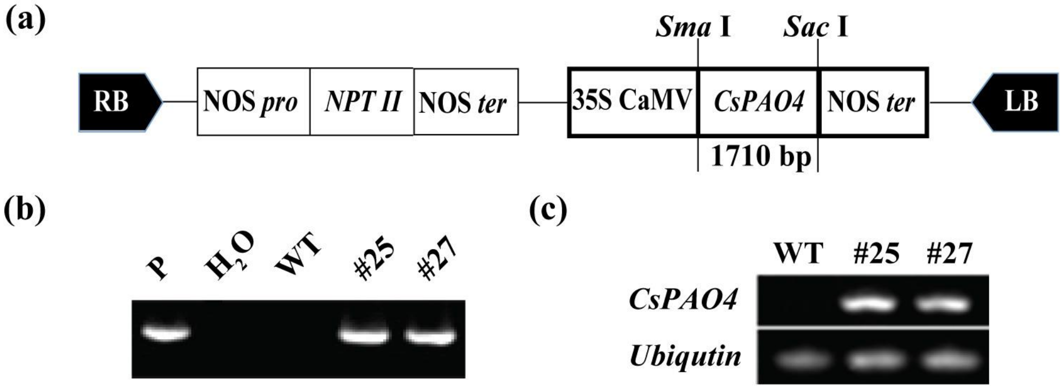
(a)
Sma I
Sac I
RB
NOS pro
NPT II
NOS ter
35S CaMV
CsPAO4
NOS ter
LB
1710 bp
(b)
P
H2O
WT
#25
#27
(c)
WT
#25
#27
CsPAO4
Ubiqutin

Figure S2

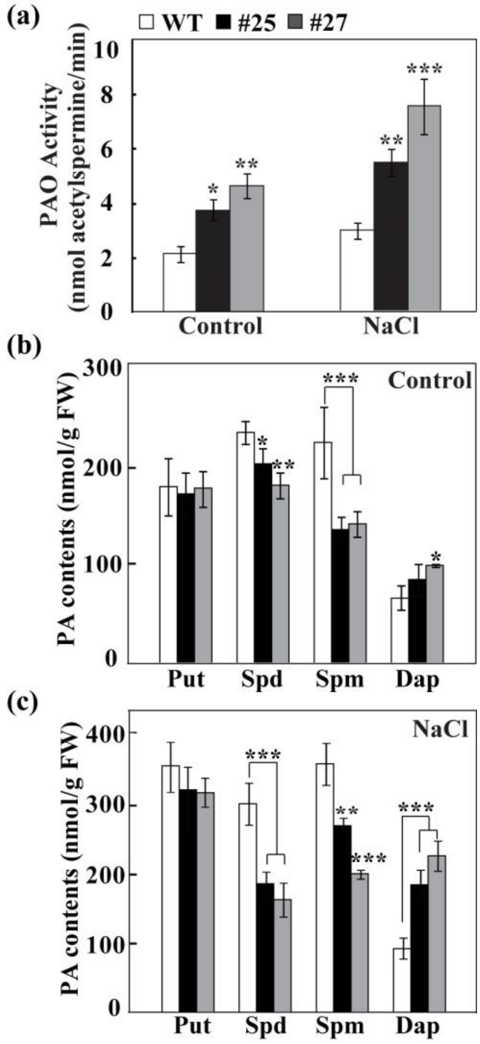
(a)
WT
#25
#27
PAO Activity (nmol acetylspermine/min)
0
2
4
6
8
10
Control
NaCl
*
**
**
***
(b)
PA contents (nmol/g FW)
0
100
200
300
Control
Put
Spd
Spm
Dap
(c)
PA contents (nmol/g FW)
0
100
200
300
400
NaCl
Put
Spd
Spm
Dap

Figure S3

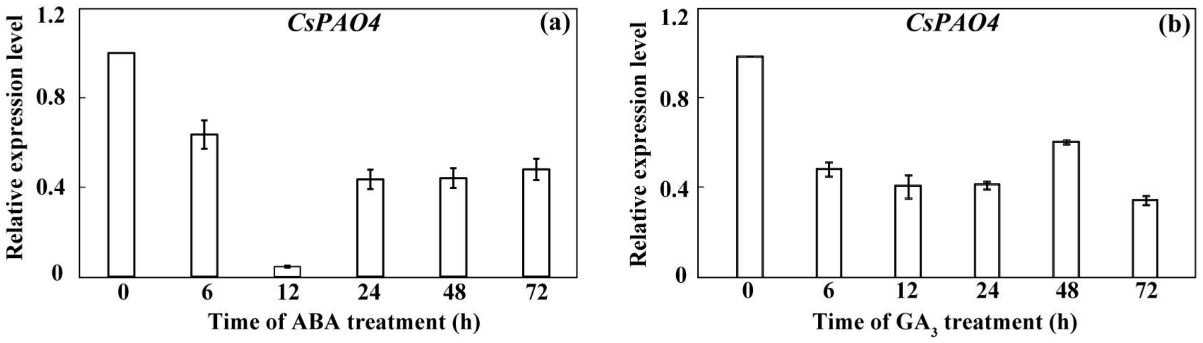
CsPAO4
(a)
Relative expression level
1.2
0.8
0.4
0
0
6
12
24
48
72
Time of ABA treatment (h)
CsPAO4
(b)
Relative expression level
1.2
0.8
0.4
0
0
6
12
24
48
72
Time of GA3 treatment (h)

Figure S4

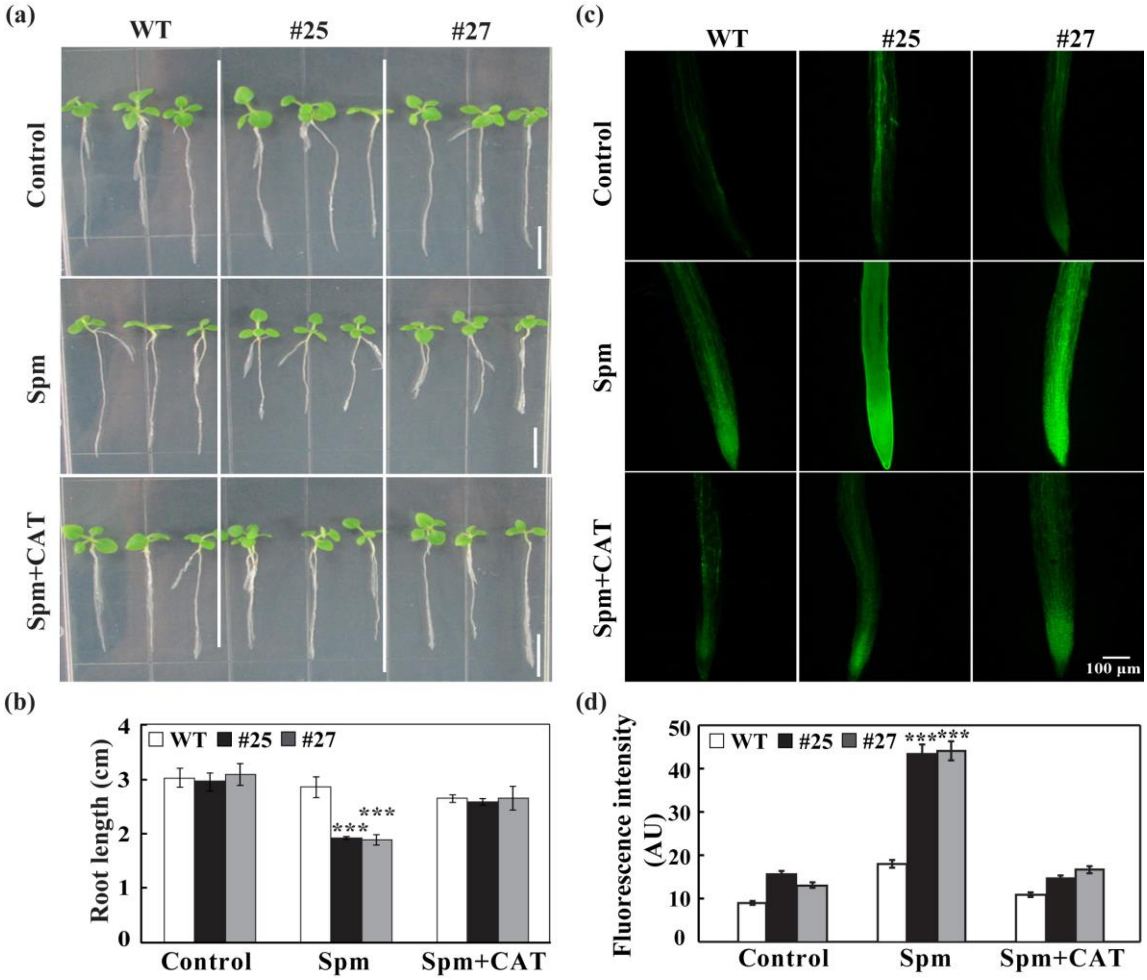
(a)
WT
#25
#27
Control
Spm
Spm+CAT
(c)
WT
#25
#27
Control
Spm
Spm+CAT
100 μm
(b)
WT
#25
#27
Root length (cm)
0
1
2
3
4
***
***
Control
Spm
Spm+CAT
(d)
WT
#25
#27
Fluorescence intensity (AU)
0
10
20
30
40
50
******
Control
Spm
Spm+CAT

Figure S5

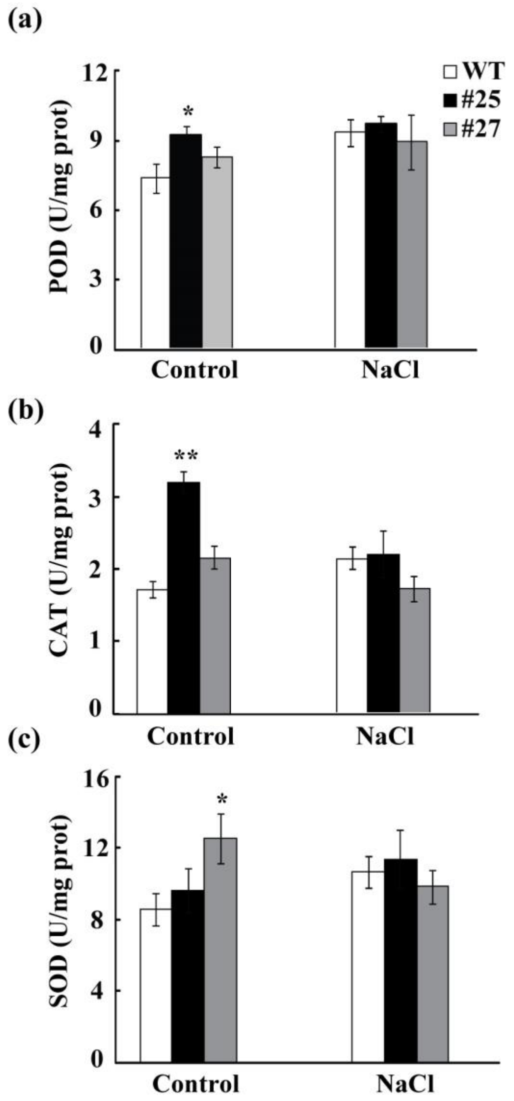
(a)
WT
#25
#27
12
9
6
3
0
POD (U/mg prot)
*
Control
NaCl
(b)
4
3
2
1
0
CAT (U/mg prot)
**
Control
NaCl
(c)
16
12
8
4
0
SOD (U/mg prot)
*
Control
NaCl

Figure S6

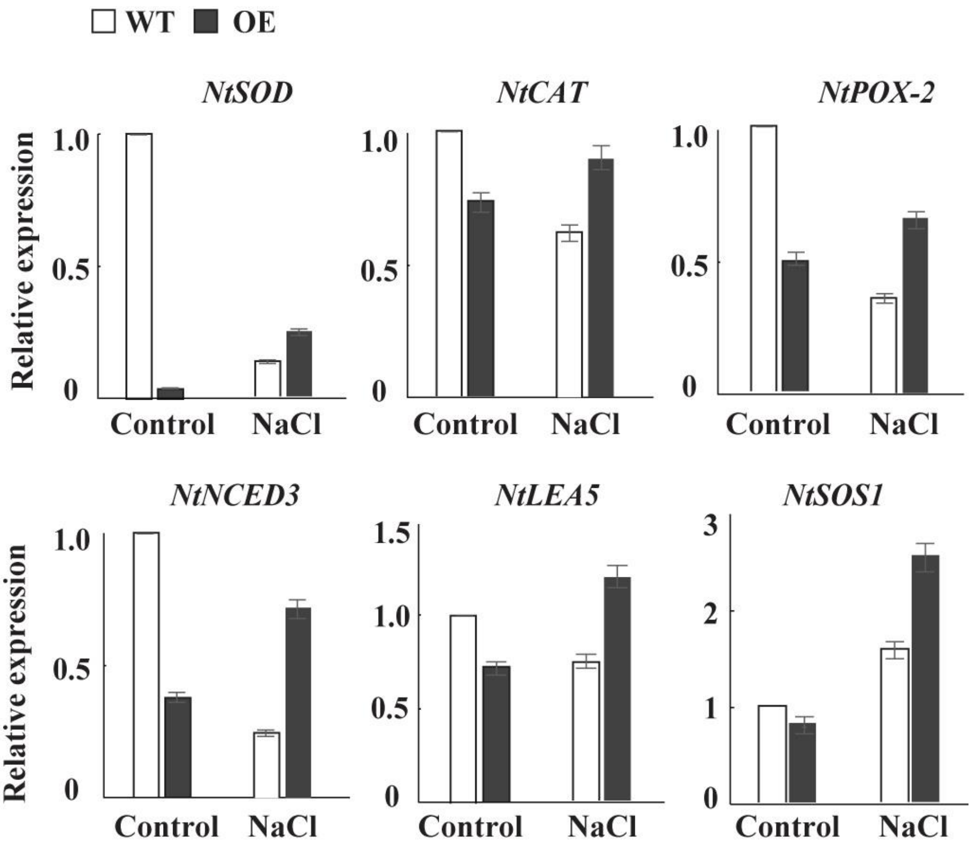
WT
OE
NtSOD
NtCAT
NtPOX-2
NtNCED3
NtLEA5
NtSOS1
Relative expression
1.0
0.5
0
1.5
3
2
1
Control
NaCl

Figure S7

Table S1. Primers used for real-time qRT-PCR and full-length PCR amplification

Primer names	Primer sequence (5’-3’)		Product length (bp)
	Forward	Reverse	
ACsPAO4$_{F/R}$	TGGCTAAGAAGCCAAGA	CTAAGCCCC CACAGAATG	1710
CCsPAO4$_{F/R}$	CAACCCGGGATGGCTAAGAAGCCAAGA	ATAGAGCTC CTAAGCCCCCACAGAATG	1728
SCsPAO4$_{F/R}$	GGACCCCCACCCACGAGGA	GCAACGGTCGTACGCTGGC	481
QCsPAO4$_{F/R}$	GCTGGCACAAGAATTGGTGGGAG	GCAACGGTCGTACGCTGGCTA	191
LCsPAO4$_{F/R}$	GCGAGGCCTGCTAAGAAGCCAAGA	AGTACGCGT AGCCCCCACAGAATG	1728
Nt SOD$_{F/R}$	AGCTACATGACGCCATTTCC	CCCTGTAAAGCAGCACCTTC	134
Nt POX$_{F/R}$	CTTGGAACACGACGTTCCTT	TCGCTATCGCCATTCTTTCT	126
NtCAT$_{F/R}$	AGGTACCGCTCATTCACACC	AAGCAAGCTTTTGACCCAGA	146
NtNCED3$_{F/R}$	AAGAATGGCTCCGCAAGTTA	GCCTAGCAATTCCAGAGTGG	132
NtLEA5$_{F/R}$	TTGAATCTGGGGTTTTGGTT	GGAAGCATTGACGAGCTAGG	126
NtSOS1$_{F/R}$	CAAATGTTATCCCCCGAAAGC	CGGAGAACCTGAGGAAATGTGA	164
Ubiqutin	GGTGTTTCCAGTGGCGGACG	TCCTCCCCTCAGCTACGGGGTAT	103
